# P09 Adult-onset Still’s disease onset following COVID-19 vaccination

**DOI:** 10.1093/rap/rkae117.040

**Published:** 2024-11-05

**Authors:** Liban Ahmed, Katie Feather, Nidhi Sofat

**Affiliations:** St George’s Hospital London, London, United Kingdom; St George’s Hospital London, London, United Kingdom; St George’s Hospital London, London, United Kingdom; St George’s University London, London, United Kingdom

## Abstract

**Introduction:**

Adult-onset Still’s disease (AOSD) is a systemic auto-inflammatory disease. The pathogenesis is thought to be a genetic predisposition with an environmental trigger. The triad of symptoms often described are daily fever spikes, arthritis, and a salmon-pink fleeting rash. Myalgia, pharyngitis, anorexia, nausea, and weight loss are sometimes present too. There is often a significant delay in the diagnosis of AOSD; this is due to the lack of specific biomarker, varied clinical presentation and its low prevalence.

**Case description:**

The patient is a 37-year-old female who presented aged 34 with pharyngitis, fevers, and global myalgia. Her past medical history included depression/anxiety and tonsilitis in her childhood. Her social history was unremarkable, except recent travel in the previous month to St Lucia. She was adopted. She took escitalopram, had no allergies and two days before symptom onset had the COVID-19 vaccine (AstraZeneca).

In general practice they gave her three courses of antibiotics; however, her blood tests showed a high C-reactive protein and raised white blood cells, with a predominant neutrophilia. She was referred to the ear, nose throat team (ENT). They gave her IV co-amoxiclav and dexamethasone. She had one episode of an evanescent rash. She had a chest x-ray, neck x-rays and CT neck which were all normal.

She was reviewed by the infectious disease team (ID), who aimed to rule out an infectious or malignant cause for the persistent fever.

She was re-admitted with the same symptoms and persisting inflammatory markers. She had a transthoracic echocardiogram which was unremarkable. She was discharged with NSAIDs which helped her symptoms. Two weeks later, the patient called to complain about the slow recovery. They checked her ferritin which was 2,720 initially. She was reviewed by rheumatology who advised a CT-PET, which showed an inflammatory picture with proximal synovitis and diffuse lymphadenopathy. It was necessary to get a lymph node biopsy, before considering immunomodulation. The patient’s symptoms persisted in the meantime, till she presented to A&E a month later. She was admitted at this point to expediate investigations, her lymph node biopsy showed an inflammatory picture. She was discharged with follow up under rheumatology clinic but started on steroids by ID.

The patient after one year is stable on methotrexate and anakinra. The latter is a subcutaneous injection of 100 mg.

**Discussion:**

There was a delay in the diagnosis as the patient was initially seen by a surgical team who may not consider rare rheumatological conditions in their differential. The self-limiting rash was noted but only occurred once while under the surgical team. The use of clinical disease scores, such as Yamaguchi, may have expedited the diagnosis. However, AOSD remains a clinical diagnosis.

It is important to rule out infectious and malignant causes before diagnosing AOSD. The involvement of the ID team allowed for a thorough assessment here. There are no specific tests; although ferritin is often raised, is not usually part of the routine tests done on admission and our patient only had hers checked a month after her first presentation. The hyperferritinaemia is thought to be due cytokine-induced secretion from reticuloendothelial system or liver damage.

Initial treatment is with NSAIDs, but this often is insufficient. The patient mentioned she felt best when she received the dexamethasone at presentation. She did not receive further steroids until much later.

There is literature to suggest vaccines such as the flu vaccine have caused new onset disease or a flare of AOSD. COVID-19 mRNA vaccines could potentially trigger similar mechanisms by autoimmunity. Conversely, there are reported cases of AOSD after COVID-19 infection. This shows that there are shared pathogenic pathways between the diseases, with interleukin-1 playing a prominent role. There is likely an induction of an aberrant innate immune system. The SARS-CoV-2 spike protein leads to cytokine storms via TLR signalling as it acts as pathogen associate molecular protein. The incidence of AOSD is low but COVID-19 and its numerous sequalae prevalent. The MHRA yellow card reporting system shows there have been 103 autoimmune disorders following AstraZeneca vaccination; however, this does not mean they have been proven to be related to the vaccine.

**Key learning points:**

• There needs to be a push to improve knowledge and awareness of the disease. Early treatment can attenuate the disease process and avoid catastrophic complications such as macrophage activation syndrome. It has diagnostic challenges and this case highlights how it requires a multi-disciplinary approach and investigations. We must encourage our colleagues to consult each other early to avoid diagnostic delay.

• Pyrexia of unknown origin is a difficult clinical scenario; however, clinicians should keep their differentials broad and involve specialists early. It would be wise to consider differentials in broad categories of infective, inflammatory, neoplastic and other. Therefore, involving rheumatology in a patient with persistent fevers and musculoskeletal systems would be pertinent as in this case. It is best to avoid empirical antibiotics without a clear source of infection. Returning travellers with fever often have non-tropical disease causes for their fever so the basics must not be missed.

• Vaccination for COVID-19 should still be recommended, as it is the best way to protect against infection and the benefits still outweigh potential risks.

• There needs to be a push for further trials in the treatment of AOSD; this will likely need to be international to have enough cases. There are no randomised controlled trials comparing different treatments, with most evidence based on case series. New treatment options could include JAK inhibitors which have been promising in mouse models.

